# Put on your thinking cap: G-quadruplexes, helicases, and telomeres

**DOI:** 10.18632/aging.100307

**Published:** 2011-04-11

**Authors:** Robert M. Brosh

**Affiliations:** Laboratory of Molecular Gerontology, National Institute on Aging, NIH, NIH Biomedical Research Center, Baltimore, MD 21224, USA

Understanding how G-quadruplex (G4) DNA structures that form in G-rich tracts of the genome affect chromosomal stability and processes such as copying the genetic information (DNA replication) or decoding the information (RNA transcription) has posed a significant challenge to researchers in the field. Although historically there has been some controversy over the existence of G4 DNA structures in vivo, emerging evidence suggests that they are indeed likely to form and have cellular consequences. In a recent study, Smith et al. investigated a role of G4 DNA in telomere capping [1], i.e., the adaptation of a nucleoprotein structure that prevents the chromosomal DNA ends from being recognized as DNA breaks and protects them from becoming degraded or fused. Telomere capping is a fairly complex process since a number of proteins have been shown to bind telomeric single-stranded or double-stranded DNA at the chromosome end. Moreover, the ability of telomeric DNA to form a variety of conformations including t-loops and G-quadruplexes adds to the complexity of how competing proteins and DNA structures influence the structural topology and metabolism of chromosome ends [2]. Using genetic and pharmacological approaches, Smith et al. showed that under conditions that stabilize G4 DNA structures, which form from a guanine-rich telomeric ssDNA exposed in a yeast temperature sensitive cdc13-1 mutant, telomere capping is in turn enhanced and phenotypes associated with capping defects are suppressed [1]. Conversely, telomere uncapping occurs under conditions that dissuade the formation of telomeric G4 DNA in the cdc13-1 mutant. The authors proposed a model in which G4 DNA structure enables G4 DNA binding proteins to further stabilize the telomere end by binding to G-quadruplex DNA, thereby preventing 5′ to 3′ exonucleolytic resection when the normal protein that blocks telomeric end processing is defective. Rad53-mediated checkpoint activation is also dampened, permitting suppression of the growth defects characteristic of the cdc13-1 mutant at the nonpermissive temperature.

Collectively, the work of Smith et al. provides strong experimental evidence that G-quadruplex-based telomere capping can help the cell evade adverse biological effects of a deficiency in natural full telomere capping. Demonstration that G-quadruplex-based telomere capping occurs in vivo is not only enlightening for telomere biologists, but more generally for those interested in the relative importance placed on noncanonical DNA structures, such as G4, in cellular nucleic acid metabolism. Traditionally, it was proposed that G-quadruplexes, which form from transient single-stranded species that arise during normal cellular DNA metabolism, may impede replication or transcription, and become a source of genomic instability. The potential of G-rich chromosomal regions to form quadruplexes was proposed to influence events not strictly limited to telomeres but also for example, certain G-rich promoter elements, ribosomal DNA repeats, and sequences in immunoglobulin heavy chain switch regions prone to rearrange [3]. Chromosomal instability of certain genetic disorders (Werner syndrome (WS), Bloom's syndrome (BS), Fanconi Anemia Group J) that are defective in DNA helicases, which like yeast Sgs1 were shown to efficiently unwind G-quadruplex DNA substrates in vitro, suggested that defects in G4 metabolism contribute to the molecular pathology of these diseases [4]; however, further evidence is required to understand the defects responsible for the clinical symptoms and cellular phenotypes. In the current study from F. Brad Johnson's lab, it was reported that deletion of the Sgs1 helicase, which belongs to the same RecQ family of DNA helicases as that of the WS helicase (WRN) or BS helicase (BLM), rescues the temperature sensitive phenotypes of cdc13-1 [1]. This effect is likely to be a consequence of the absence of Sgs1 helicase action on the telomere-associated G4 structures because expression of Sgs1 proteins that are defective in helicase activity or lack the RQC domain that binds G4 DNA with high affinity negates the ability of Sgs1 to promote telomere uncapping in the cdc13-1 mutant. At first glance, the findings for a role of Sgs1 in telomere uncapping seem counterintuitive especially in light of the suggestion that Sgs1, WRN, or BLM helicases help to preserve chromosomal stability at G-rich sequences by resolving G-quadruplex DNA structures. This logic may be too simplistic, as suggested by the study from the Johnson lab, and also consistent with other recent demonstrations that Sgs1 stimulates 5′-3′ exonucleolytic resection of DNA breaks and telomere ends [5-9]. Careful experimentation with human cells will be required to unveil the potential roles of WRN and BLM helicases in G4 DNA metabolism that are likely to be context specific. WRN is implicated in telomere lagging strand synthesis [10] and associated with telomeres in human cells that undergo alternative lengthening of telomeres (ALT) [11]. BLM is required for suppression of telomere fragility [12]. Both WRN and BLM interact with telomere associated proteins (for review, see [13]). Since mounting evidence suggests that G-quadruplexes form at telomeres, a direct role of WRN and BLM helicases in the metabolism of these structures is plausible.

Not to lose sight of a significant conclusion from the Smith et al. publication, it seems that G-quadruplex formation and stabilization may have unexpected biological outcomes and not always deleterious consequences. Along these lines, other recent work from the Johnson lab showed that in WS and BS mutant cells, upregulated genes are significantly enriched for predicted G-quadruplex forming sequences [14]. This finding goes against the concept that G4 structures impede transcript production by RNA polymerase. A provocative idea raised by Johnson et al. is that G4 structures may exclude nucleosomes or other duplex DNA binding proteins that would otherwise interact with the more conventional B-form double helical DNA and repress transcription [14]. Further work in this area is necessary since recent studies suggest that G-rich DNA sequences impede transcription in vitro. Depending on the DNA template sequence and reaction conditions, transcription blockage may occur due to formation of unusually stable RNA/DNA hybrids or triplexes by guanine-rich DNA sequences [15], in addition to G-quadruplexes. Adding to all this complexity, sgs1 deficiency in yeast was shown to exert a downregulatory effect on expression of genes that contain sequences with G4 forming potential [16], opposite to that observed in cells from WS and BS patients. Genome architecture, other protein factors involved in the metabolism of G-rich sequences, or mechanisms of action of DNA helicases may contribute to the observed differences in gene regulation between yeast and human. Furthermore, because G4-DNA is actually a family of structures, the distributions of particular G4-DNA folds may differ across organisms or genomic sites.

It is conceivable that differences in substrate specificity between G4 helicases target the enzymes to distinct G4 structures with alternate configurations. G4 can be intramolecular or intermolecular, and the orientation of the G-rich strands engaged in Hoogsteen hydrogen bonding or the nature of the intervening loops may confer differential effects on the activity of proteins that selectively bind and/or unwind the G4 structure [17]. This area of investigation is at its infancy; however, one known difference in the behavior among the known G4 DNA helicases is their polarity of unwinding (for review, see [4]). WRN and BLM, as well as G4R1 (personal communication, S. Akman and J. Vaughn), the latter of which is thought to be involved in RNA processing [18;19], are 3′ to 5′ helicases, whereas the sequence-related FANCJ and Pif1 proteins are 5′ to 3′ helicases. Interestingly, a newly discovered member of the FANCJ/Pif1 family is the ChlR1 helicase genetically linked to Warsaw Breakage Syndrome, a chromosomal instability disorder characterized by cohesion defects and sensitivity to DNA cross-linking agents [20]. It is not yet known if ChlR1 helicase is active on G-quadruplex DNA structures; however, the protein has been shown to be active on duplex DNA [21;22]. Additional members of the FANCJ family of DNA helicases include mouse RTEL and nematode DOG-1, which have been implicated in telomere maintenance [23] and stability of sequences flanking G-tracts [24], respectively; however, neither of these proteins has been shown to have helicase activity on secondary structures in G/C tracts.

An important priority is to ascertain if the clinical and cellular phenotypes of the known genetic helicase disorders are at least partly due to aberrant metabolism of structures that form in G-rich tracts, and the underlying mechanisms for the peculiar forms of chromosomal instability characteristic of the diseases. FANCJ-depleted human cells are sensitive to a G4-specific binding compound and show elevated DNA damage and apoptosis upon exposure to the drug [25]. Moreover, FANCJ-deficient cells accumulate deletions at genomic sequences with a G4 DNA signature [26], suggesting that FANCJ prevents replication-associated DNA damage by removing G4 structures. Although a biological function of PIF1 in mammalian cells has not yet been determined, yeast Pif1 is involved in the maintenance of nuclear and mitochondrial genome stability [27]. Sequences predicted to form G4 structures are enriched in the mitochondrial genome [28], but a specialized role for a helicase in the resolution of mitochondrial G4 structures has not yet been revealed. Recent work from the Nicolas lab showed that Pif1 prevents genomic instability of a G4 forming human minisatellite sequence inserted into the S. cerevisiae nuclear genome [29]. Pif1 helicase is also involved in the coordination of checkpoint activation following telomere uncapping [30]. As a 5′ to 3′ helicase, the mechanism of action of Pif1 on DNA structures at the telomeric end is likely to be different from that of the 3′ to 5′ Sgs1 helicase. In addition to these helicases, the Dna2 helicase-nuclease implicated in Okazakai fragment processing, and thought to have a role in telomere maintenance, unwinds G-quadruplex substrates with a 5′ ssDNA tail, but can also degrade G-quadruplexes in the presence of the single-stranded DNA binding protein RPA [31]. It is plausible that Dna2 may help to process G-quadruplex structures that form during lagging strand DNA synthesis.

Clearly, there is much work to do to understand the significance and fate of G4 structures believed to exist at telomeres or other G-rich regions of the genome. The growing number of helicases implicated in the maintenance of genomic stability and shown to unwind G4 nucleic acids suggests multiple layers of complexity in terms of helicase involvement. A large family of G4 structures exists and the subgenomic regions that G4 structures form or persist during specific phases of the cell cycle may be important. While it is tempting to speculate that unwinding of G-quadruplexes preserves genomic stability, it may be that in defined genetic mutant backgrounds, the action of G4 DNA helicases have detrimental effects on genome homeostasis. This is highly relevant since a number of genetic disorders characterized by genomic instability and/or sensitivity to agents that impose DNA damage or replication stress are attributed to deficiency in function of a gene that encodes a protein that may affect the formation or stability of G-quadruplexes. In the quest to solve the mystery of genome preservation, it should not be forgotten that DNA structures as well as the proteins that bind or metabolize these structures may coordinately act to protect chromosomes from insult or decline under certain cellular conditions.
